# CIITA methylation and decreased levels of HLA-DR in tumour progression

**DOI:** 10.1038/sj.bjc.6602045

**Published:** 2004-07-27

**Authors:** N Iizuka, M Oka

**Affiliations:** 1 Departments of Surgery II, Yamaguchi University School of Medicine, 1-1-1 Minami-Kogushi, Ube, Yamaguchi 755-8505, Japan

**Sir**,

We read with interest the article of Morimoto *et al* (2004) that investigated the relation of epigenetic inactivation of *CIITA* with levels of *HLA-DR*, one of the MHC class II genes, in haematopoietic tumour cells.

Recent DNA microarray studies have showed that decreased levels of MHC class II molecules are associated with high metastatic potential and/or poor prognosis, not only in haematopoietic tumour (Rimsza *et al*, 2004) but also in several adenocarcinomas (Ramaswamy *et al*, 2003) and hepatocellular carcinoma (HCC) (Iizuka *et al*, 2003), suggesting the common role of MHC class II molecules in tumour progression. In this regard, elegant work by Morimoto *et al* (2004) could provide a therapeutic strategy against various malignancies. The authors stated that, by suppressing the expression of MHC class II molecules, epigenetic inactivation of CIITA provided a survival advantage to a subset of haematopoietic tumours (Morimoto *et al*, 2004); however, they did not investigate the relation between levels of *CIITA* and *HLA-DR* and tumour progression.

Our recent DNA microarray study showed that decreased levels of *HLA-DRA* gene were related to recurrence of HCC (Iizuka *et al*, 2003). We also confirmed that levels of HLA-DR protein by tumour cells were related to recurrence of HCC (unpublished data). Using the DNA microarray data set of HCC (available at http://surgery2.med.yamaguchi- u.ac.jp/research/DNAchip/hcc-r ecurrence/index.html), we investigated the relation between levels of *CIITA* (three probes, U18288, U18259, and X74301) and those of *HLA-DRA* (probe X00274) and *HLA-DRB1* (probe M33600). Given that *CIITA* levels in HCC were markedly low, it is reasonable to assume that this phenomenon may be due in part to its epigenetic inactivation. However, in our data, there were no correlations between levels of *CIITA* and those of the two *HLA-DR* genes. Thus, in the context of human cancer tissues, the transcriptional regulation of *HLA-DR* is likely to be much complex. From this standpoint, we recommend that the authors will clarify the relation of *CIITA* methylation status to constitutive expression of *HLA-DR* in a larger cohort of haematopoietic tumour. On the basis of their finding (Morimoto *et al*, 2004) and our present finding, in our opinion, methylation status of *CIITA* could be a therapeutic target combined with interferon-gamma for preventing the progression of haematopoietic tumour and *HLA-DR* may be superior to *CIITA* as a marker for progression of haematopoietic tumour.

Additionally, tumour HLA-DR might have a function other than the antigen-presenting function. Recently, Altomonte *et al* (2003) showed a possible involvement of HLA-DR in a signalling pathway linked to cell adhesion in melanoma cells. Thus, we expect that further work by Morimoto *et al* would provide a clue to incorporate MHC class II antigens into the mainstream of molecular basis underlying tumour progression.

## References

[bib1] Altomonte M, Fonsatti E, Visintin A, Maio M (2003) Targeted therapy of solid malignancies via HLA class II antigens: a new biotherapeutic approach? Oncogene 22: 6564–6569 14528281 10.1038/sj.onc.1206960

[bib2] Iizuka N, Oka M, Yamada-Okabe H, Nishida M, Maeda Y, Mori N, Takao T, Tamesa T, Tangoku A, Tabuchi H, Hamada K, Nakayama H, Ishitsuka H, Miyamoto T, Hirabayashi A, Uchimura S, Hamamoto Y (2003) Oligonucleotide microarray for prediction of early intrahepatic recurrence of hepatocellular carcinoma after curative resection. Lancet 361: 923–929 12648972 10.1016/S0140-6736(03)12775-4

[bib3] Morimoto Y, Toyota M, Satoh A, Murai M, Mita H, Suzuki H, Takamura Y, Ikeda H, Ishida T, Sato N, Tokino T, Imai K (2004) Inactivation of class II transactivator by DNA methylation and histone deacetylation associated with absence of HLA-DR induction by interferon-gamma in haematopoietic tumour cells. Br J Cancer 90: 844–852 14970863 10.1038/sj.bjc.6601602 PMC2410180

[bib4] Ramaswamy S, Ross KN, Lander ES, Golub TR (2003) A molecular signature of metastasis in primary solid tumors. Nat Genet 33: 49–54 12469122 10.1038/ng1060

[bib5] Rimsza LM, Roberts RA, Miller TP, Unger JM, LeBlanc M, Braziel RM, Weisenburger DD, Chan WC, Greiner TC, Muller-Hermelink HK, Jaffe ES, Gascoyne RD, Campo E, Fuchs DA, Spier CM, Fisher RI, Delabie J, Rosenwald A, Staudt LM, Grogan TM (2004) Loss of MHC Class II gene and protein expression in diffuse large B cell lymphoma is related to decreased tumor immunosurveillance and poor patient survival irrespective of other prognostic factors: a follow-up study from the Leukemia and Lymphoma Molecular Profiling Project. Blood 103: 4251–4258 14976040 10.1182/blood-2003-07-2365

